# The relationship between the built environment and habitual levels of physical activity in South African older adults: a pilot study

**DOI:** 10.1186/s12889-015-1853-8

**Published:** 2015-05-30

**Authors:** Tracy L. Kolbe-Alexander, Kyla Pacheco, Simone A. Tomaz, David Karpul, Estelle V. Lambert

**Affiliations:** Department of Human Biology, UCT/MRC Research Unit for Exercise Science and Sports Medicine, Faculty of Health Sciences, University of Cape Town, Cape Town, South Africa; Centre for Research on Exercise, Physical Activity and Health, School of Human Movement and Nutrition Sciences, University of Queensland, Blair Drive St Lucia, 4072 Brisbane, Australia

**Keywords:** Built environment, Physical activity, Older adults

## Abstract

**Background:**

Previous research has shown that the built environment plays a role in habitual levels of physical activity (PA), however much of this research has been conducted in adults and higher income countries. The aim of this pilot study was to examine the strength of association between the built environment and PA in South African older adults.

**Methods:**

Participants were recruited (*n* = 44, mean age 65 ± 8.5 years) from two suburbs, representing either a high socioeconomic (HSA) or low socioeconomic area (LSA). Self-reported PA, and subjective assessments of neighborhood walkability (Neighborhood Environment Walkability Scale, NEWS) was measured. Participants wore Actigraph GT3x accelerometers to objectively quantify PA.

**Results:**

HSA participants reported significantly more leisure-time and less transport PA. Objectively measured and self-reported MVPA was significantly higher in HSA participants. NEWS ‘Land-use Mix’ was negatively associated with leisure-time MVPA, (r^2^ = 0.20; *p* < 0.02). In addition, neighborhood aesthetics was positively associated with leisure-time physical activity (r^2^ = 0.33; *p* = 0.02). ‘Safety from traffic’ was inversely associated with travel-related PA (r^2^ = 0.14, *p* = 0.01). None of the other NEWS scores were associated with PA for the total group.

**Conclusion:**

Leisure-time and transport-related PA was influenced by socio-economic status. Attributes of the perceived built environment associated with leisure-time and total MVPA in older South Africa adults were different in low- and high- income settings.

## Background

The average population of Africa is growing older and the projected shift suggests that the number of adults reaching 60 years or more is expected to double in many African countries in the next 50 years [1]. It is anticipated that the South African older adult population will increase by 300 % from 1985–2025 [2]. The increase in the proportion of persons older than 60 years is one of the contributing factors to the increased burden of non-communicable diseases (NCD) [2]. Recent data shows that the prevalence of self-reported NCD among South African older adults is 52 % with 23 % have more than one NCD [3].

There is consistent evidence of association between physical activity and reduced risk for non-communicable diseases [4]. Even in older adults, regular physical activity has been associated with an overall reduction in morbidity and mortality [5, 6]. Regular physical activity in older adults can help increase functional independence [7], increase mobility and muscle strength [7, 8], maintain glucose blood levels [9], improve bone health and bone mineral density [7, 9], improve psychological [9] well-being as well overall quality of life [7, 10].

Despite the evidence of the benefits of regular physical activity, there is a trend for activity levels to decline with increasing age [6, 11]. Indeed, the prevalence of sufficiently active men and women declines from 34 % in 15–24 year olds to 7 % among those older than 65 years [12]. The decline in activity levels is more pronounced in women, low-income groups and in persons with low education levels [13]. Specific barriers to physical activity in older adults have been identified and include: poor health, reduced mobility and fear for personal safety due to crime and traffic [6]. For example, Amosun et al. [14] found that older adults were unable to walk while carrying packages, at a pace that would allow them to cross at an intersection in the time taken between a change in traffic signals. Furthermore, more than half of them felt apprehension, anxiety or fear while crossing roads [14].

The link between attributes of the built environment and physical activity, such as mixed land use and street connectivity, has been well described in adult populations [15]. There are also studies demonstrating that the location and nature of recreation facilities, shopping and transit destinations in a neighborhood may also influence levels of physical activity, and walking in particular. Sallis et al. found that access to low cost recreation facilities and the presence of sidewalks on most streets were significantly associated with meeting the physical activity guideline of 150 min of moderate-vigorous intensity PA (MVPA) per week, OR = 1.16 and 1.47, respectively among adults between the ages of 18 and 65 years [16]. Conversely, Gomez et al., [17] reported a negative association between street connectivity and walking among older adults living in Bogata, Columbia. Thus the influence of the built environment, including forms of residence and location of retail outlets, on physical activity and walking may differ from country to country, and in persons in different age groups [10]. Areas with high residential density and street connectivity together with land-use mix have been used to determine the ‘walkability’ of neighborhoods [18].

The construct of “walkability” of a neighborhood and the relationship between the perceived neighborhood environment and physical activity levels is an important one, as it may provide insights for urban planners and policy makers as to how the built environment, and aspects of safety and zoning, traffic calming and shopping, may impact on health-seeking behaviours such as physical activity. However, much of the research has been conducted in the Global North, with some evidence from South America [10]. In addition, there is limited research among older adults, as most studies investigating the relationship between the built environment and physical activity have been conducted amongst adults younger than 50 years of age [10].

Thus, there is relatively little data on the relationship between perceived neighborhood environment and PA from countries from the Global South and virtually no data amongst older adults. Therefore, the aim of this research study was to estimate strengths of association between measures of the built environment and habitual levels of physical activity in South African older adults.

## Methods

### Setting

Two suburbs were selected for this cross-sectional pilot study, where one suburb represented a high socioeconomic area, Claremont (HSA) and the other a low socioeconomic area, Athlone (LSA). The socioeconomic status of the areas was based on average housing prices according to the March 2012 report which were obtained from the “Property 24 Cape Town” website (http://property24.com/for-sale/cape-town/western-cape/432). Table 1 compares the demographic data from these two suburbs [19] (City of Cape Town 2011 Census; https://www.capetown.gov.za/en/stats/Pages/2011-Census-Suburb-Profiles-land.aspx). These suburbs were comparable as they are largely residential, but both have a main road populated with commercial shops representing mixed land use. Both have schools in the area. Therefore the Claremont and Athlone suburbs were selected for the study as they had similar land-use mix, both had an exercise group for older adults, and but had different socio-economic status. Eligible participants lived within 5 km of a recreational facility and this buffer was used as the criterion to define their neighborhood in order to include destinations that are accessible by walking and motorised transport.

**Table 1:** Demographic characteristics of the Athlone (LSA) and Claremont (HAS) suburbs [19]

Variable	LSA	HSA
Average household size *(number of people)*	3.8	2.5
Formal Dwelling (%)	95	99
Education *(Completed high school or tertiary-%)*	59	90
Unemployment rate (%)	12	4
Household income		
< ZAR 3 200 per month (%)	31	10
> ZAR 102 401 per month (%)	2	12

### Participants

A convenience sample (*n* =44) of men and women older than 50 years participated in the research study. The participants living in the LSA were recruited from a community-based exercise program for older adults (Community Health Intervention ProgrammeS, CHIPS, Live it Up group [20] and by word of mouth. Similarly, those living in the HSA were recruited from members of the Sports Science Institute of South Africa’s (SSISA) group exercise classes, and by word of mouth. This is important as recruitment in both groups represented, at least in part, members of group-based exercise programs which comprised walking and low impact circuit exercises.

### Inclusion and exclusion criteria

Men and women 50 years old or older and living within 5 km of the CHIPS programme site or SSISA were eligible to participate in the research study. Older adults that had been previously diagnosed with a stroke, Parkinson’s disease, balance disorders, impaired vision or had uncontrolled conditions such as hypertension or diabetes (not on medication) were not eligible to participate.

## Measures

### Questionnaires

#### Demographic variables

The researchers administered a socio-demographic questionnaire, which included questions such as age, gender, education, marital status and vehicle ownership. These questions were comparable to that of Hanibuchi et al., [10] who conducted a similar research study among Japanese older adults. In addition, participants reported on whether they used public transport, which included the bus, train or mini-bus taxis. The minibus taxis carry up to 15 passengers each, and is one of the most accessible modes of public transport in South Africa [21].

#### Self-perceived neighborhood environment

Participants completed the Neighborhood Environment Walkability Scale (NEWS) which assesses perceived residential density, land use mix - diversity, land use mix – access, walking/biking infrastructure, aesthetics and traffic and crime safety [22].

### Residential density

Residential density is based on the number and types of various residences like detached single-family residences, townhouses and apartments in the neighborhood.

### The land-use Mix

Diversity score is based on the perceived proximity of 23 different destinations from home, ranging from less than 5 min walk to more than a 30 min walk from home [23]. A 5-point likert scale was used to score the proximity of destinations where a score of 5 was given to destinations closest to home and a score of 1 allocated to destinations further from home. The mean value the 23 responses were recorded as the Land-use Mix Diversity score, with a higher score representing more facilities and destinations close to home.

Participants rated the number of destinations within walking distance from their home using a 4-point Likert scale, ranging from strongly disagree (1) to strongly agree (4) to calculate the Land-use Mix Access score. The average score for the seven questions was recorded as the Land-use Access score, with a higher score indicating more destinations within a 10–15 min walk from home.

### Street connectivity

Similarly, a Likert scale was used to measure the Street Connectivity, which was based on the number of *culs-da-sac* and intersections in the neighborhood . A score of 4 was allocated to responses where the participant ‘strongly agrees’ and a score of 1 where they ‘strongly disagree. The higher the score, the better the street connectivity.

### Walking/cycling facilities

Walk/Cycle Infrastructure score was based on the presence of sidewalks, pedestrian and cycle trails and physical barriers between walk/cycle paths and roads. The scoring was based on a 4-point likert scale as for ‘Street Connectivity’. A higher score implies greater infrastructure and facilities to promote walking and cycling.

### Aesthetics/neighbourhood surroundings

The presence of trees, attractive sights and litter were some of the items in the Aesthetics score. Participants reported their level of agreement with six statements, ranging from strongly agree to strongly disagree. The average score was recorded, with scores ranging from 1 to 4, and a higher score suggests that neighborhood is more aesthetically pleasing.

### Safety from traffic and crime

Safety from traffic and crime were also scored on the 4-point Likert scale and included items where participants reported on volume and speed of traffic, availability of pedestrian crossings and perceived crime rates. The closer the average score is to 4, the safer the participants feel from traffic, whereas a lower score suggests that they feel less safe. Safety from Crime was calculated in the same way for the questions related to crime.

### Neighbourhood satisfaction

Lastly, the neighborhood Satisfaction score was the average of 17 items scored using a 5-point likert scale described in the other sub-scores. The mean of the 17 items were recorded and a higher score (closer to 5) suggests greater satisfaction.

### Habitual levels of physical activity

#### Self-reported physical activity

The Global Physical Activity Questionnaire (GPAQ) was used to collect data on self-reported physical activity [24]. The GPAQ includes questions on habitual levels of light, moderate and vigorous intensity physical activity. The physical activity domains include work-related activity, transport and leisure-time activity. Outcome measures included work-related moderate-vigorous intensity physical activity (MVPA), transport-related MVPA, leisure time MVPA and total MVPA. Total MVPA was used to determine if participants met the physical activity guideline of 150 min or more, per week.

### Objective measures

Participants were requested to wear the Actigraph GTX3 accelerometer for 7 consecutive days [25]. A minimum of 4 days of data with at least 600 min (10 h) of data per day was required for statistical analysis [26]. Light, moderate and vigorous intensity physical activity was calculated from cut-points previously described by Matthews et al., [25]. Counts less than 759 per minute were categorised as light intensity physical activity, those between 760 and 5998 per minute were moderate intensity and counts more than 5999 per minute were categorised as vigorous intensity physical activity. Total time for sedentary, light, moderate and vigorous intensity was calculated.

### Statistical analysis

The STATA software package was used for all the analyses. Mean, standard deviation and standard error were calculated for the continuous variables, which included demographic information, physical activity (GPAQ, accelerometer and pedometer counts). Frequency tables were computed for categorical variables. T-tests for independent groups and Chi^2^ analyses were used to determine significant differences between older adults from the higher and lower socio-economic areas.

Spearman’s correlation coefficients were used to characterize the relationship between the perceived neighborhood environment and physical activity. Furthermore, participants were categorised as physically active if they met the guideline of 150 min of MVPA per week, and inactive if they did not.

### Ethical considerations

Each participant received ZAR 50 (~4.65 USD) for the time taken to complete the questionnaires. There were minimal risks associated with participating in this research study because participants were asked to maintain their habitual levels of daily activity. Informed written consent was obtained from all participants prior to entry into the study. This research study was approved by the UCT Health Sciences Research Ethics Committee (HSREC REF 168/2012).

## Results

### Participant characteristics

The participants’ mean age was 65.0 ± 8.5 years. The total sample comprised of 10 men and 34 women, with HSA having significantly more men than the LSA group (Table 2). The HSA residents had significantly higher levels of education than those in the LSA with all but one resident having a secondary level education or higher (chi^2^ = 26.22; *p* < 0.001) (data not shown). Just over a third (36 %) of the participants were employed either fulltime or part time, with more participants from HSA having a job (Table 2). Furthermore, significantly more participants from the HSA received an income from investments (Table 2). The main source of income for those living in LSA was the state pension, and this was significantly more than for the HSA residents.

**Table 2:** Participant Characteristics

Variable	Total	LSA	HSA
	(*n* = 44)	(*n* = 24)	(*n* = 20)
Age (years)	64.6 ± 8.5	65 ± 10	64 ± 6
Males (n; %)	10 (22)	1 (4)	9 (43)^**^
Employed (n; %)	16 (36)	5 (21)	11 (52)^*^
Pension (n; %)	19 (42)	16 (67)	3 (14) ^**^
RA (n; %)	16 (38)	9 (38)	7 (38)
Investments (n; %)	12 (27)	3 (7)	9 (43)^**^
Car ownership (n; %)	27 (62)	7 (29)	20 (100)^*^

Legend

Age: data presented as mean ± standard deviation

**: *p* < 0.01

^*^: *p* < 0.05

Total MVPA = occupation + leisure time + transport related MVPA

**Fig. 1:**
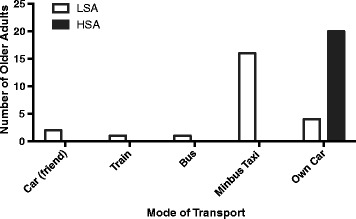
Mode of transport for LSA and HSA Participants. Pearson Chi 2 = 31.5; *p* < 0.001

All of the participants living in HSA owned a car while only 7 of those living in LSA had access to a motor vehicle at home (Table 2). Consequently, the mode of transportation was significantly different between the two groups and none of the HSA participants reported using public transport when travelling from place to place (Fig. 1). Two-thirds the LSA participants reported using a mini-bus taxi as their main form of transportation, whereas the all the HSA participants reported using their own car.

### Neighborhood environment

The NEWS Land use mix diversity score, which represents the walking time to various facilities in the neighborhood, was not significantly different between the two groups (Table 3). However, those living in HSA reported significantly more facilities that were further from home, 5.1 ± 4.3 versus 1.8 ± 3.3, p < 0.001 (data not shown). The NEWS-Land use mix access score was significantly higher for those living in LSA, and represents the number of places to visit the in the neighborhood and access to services like public transport and stores (Table 3). The HSA neighborhood was perceived to be more aesthetically pleasing than LSA (Table 3). None of the other scores obtained in the NEWS survey were significantly different between the two groups (Table 3).

**Table 3:** NEWS survey for the total sample and by neighborhood (mean ± SD)

Variable	Total	LSA	HSA
	(*n* = 44)	(*n* = 24)	(*n* = 20)
Residential Density	216.8 ± 36.4	209.6 ± 20.9	225.4 ± 48.3
Land-use Mix: Diversity	3.0 ± 0.8	3.1 ± 0.9	2.9 ± 0.7
Land-use Mix: Access	3.1 ± .04	3.3 ± 0.2	2.9 ± 0.5^#^
Street Connectivity	2.7 ± 0.6	2.6 ± 0.7	2.7 ± 0.4
Walk/Cycle Infra-structure	2.5 ± 0.6	2.6 ± 0.8	2.5 ± 0.4
Aesthetics	2.8 ± 0.7	2.5 ± 0.7	3.0 ± 0.6*
Safety from Traffic	2.2 ± 0.7	2.1 ± 0.8	2.4 ± 0.5
Safety from Crime	2.6 ± 0.5	2.5 ± 0.5	2.6 ± 0.5
Neighbourhood satisfaction	3.6 ± 0.5	3.5 ± 0.6	3.7 ± 0.5

Legend

#: *p* < 0.01

*: *p* < 0.03

### Self-reported physical activity

**Fig. 2:**
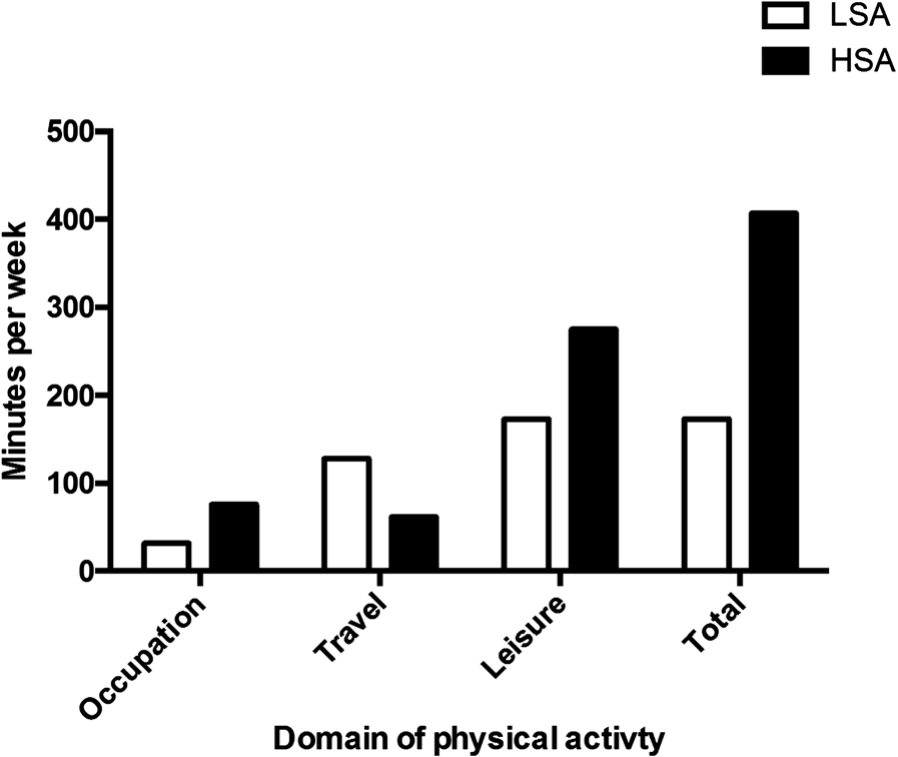
Self-reported moderate-vigorous intensity physical activity for LSA and HSA older adults. Legend: * = *p* < 0.05

Most of the participants reported more than 150 min of MVPA per week. The participants living in HSA reported significantly more weekly, moderate-vigorous intensity physical activity than those living in the LSA (Fig. 2). These participants reportedly spent significantly more time in leisure-time physical activity and significantly less time in transport-related physical activity than the LSA residents (Fig. 2). Therefore, leisure time physical activity was the main contributor to total MVPA for those living in HSA, while transport-related activity was the main contributor for LSA. There were no significant differences in reported time spent sitting between the two groups.

### Objective measures of physical activity

Data from the GT3x accelerometer is in line with that from the GPAQ questionnaire, which shows that the HSA participants had significantly greater total moderate-vigorous intensity physical activity than those from LSA (Table 4). No vigorous intensity physical activity was recorded for the LSA residents, and only 15.1 ± 39.8 min per week were recorded for HSA residents. Despite the HSA participants having higher levels of physical activity, they had significantly higher sedentary time than the LSA group (Table 4).

**Table 4:** Objectively measured physical activity in minutes per week (Median; IQR)

Variable	Total	LSA	HSA
	(*n* = 45)	(*n* = 24)	(*n* = 21)
Light intensity	2463; 2286-3086	2685; 1972-2872	2450; 2308-2798
Moderate Intensity	555; 385-830	373; 340-459	642; 465-769^**^
Vigorous Intensity	0; 0-23	0;0-0	0; 0-14
Time spent sedentary	5825; 5010-7107	5266; 4654-5744	6751; 5742-6949^**^
Total MVPA	555; 385-995	373; 340-459	644; 465–797^**^

Legend

*: *P* < 0.01

**: *p* < 0.03

### Relationship between perceived neighborhood environment and physical activity

The NEWS Land use mix access score was negatively associated with self-reported weekly leisure time MVPA for the total sample (Table 5). Therefore, leisure time MVPA is lower when there is more limited access and proximity to services and facilities such as stores and transit stops (Table 5).

**Table 5:** The relationship between perceived neighborhood environment and physical activity

	NEWS	Physical Activity	R^2^	p
All participants (*n* = 44)				
	Land-use Mix: Access	Leis MVPA^g^	0.20	0.02
	Safety from Traffic	Travel MVPA^g^	0.14	0.01
	News aethetics	Leis MVPA	0.33	0.02
LSA (*n* = 24)				
	Crime	Total MVPA	0.41	0.04
	Residentail Density	Travel MVPA^g^	0.15	0.09
	Walk/cycle infrastructure	Travel MVPA^g^	0.15	0.06
HSA (*n* = 20)				
	Aesthetics	Travel MVPA	0.30	0.009
	Safety from Traffic	Travel MVPA^g^	0.15	0.09
	Residential Density	Total MVPA^a^	0.21	0.05

Legend

G = GPAQ

A = Accelerometer

Total MVPA = occupation + leisure time + transport related MVPA

In addition, perceived safety from traffic was inversely associated with self-reported travel-related physical activity for the total sample (Table 5). This finding approached significance for the HSA group, where a higher score, suggesting less safety from traffic, was associated with lower levels of travel-related physical activity. The safer the residents in LSA felt (from crime), the higher the self-reported MVPA. The availability of walking and cycle paths were significantly and positively associated with travel related physical activity for the LSA residents R^2^ = 0.30; *p* = 0.009, (Table 5).

The only NEWs variables significantly associated with objectively measured physical activity were residential density and neighborhood satisfaction, and this was only in the HSA residents (Table 5). None of the other NEWS scores were significantly associated with self-reported or objective measures of physical activity.

## Discussion

The main aim of this pilot study was to characterize the relationship between the perceived built environment and physical activity in older adults from both low and high socio-economic neighborhoods, respectively.

The first important finding was that older adults from the LSA had significantly higher levels of self-reported active transport and lower leisure-time and total MVPA than those living in the HSA. This is supported by findings from other low-middle-income countries such as China and Brazil [18, 27]. Chinese older adults representing a higher socio-economic status participated in less walking for transport and more walking for recreation than those from the lower socio-economic groups [27]. Similarly, the prevalence of Brazilian adults from lower income areas meeting physical activity guidelines was less than those in higher income areas [18]. The Brazilian study also found that adults between the ages of 18 and 65 years, living in higher income areas reported more leisure time walking than their counterparts [18]. Therefore, it appears that neighborhood socio-economic status potentially influences physical activity in both younger and older adults living in developing and low-middle-income countries.

It is likely that the difference in transport related physical activity between the two neighborhoods in our study is due to the differences in motor vehicle ownership. None of the LSA residents had access to a motor vehicle at home and they walked twice as much for transport than the HSA residents. Likewise, Turrel et al., [28] reported that Australian adults living in low-income neighborhoods were less likely to have access to a motor vehicle at home and that there was a linear relationship between motor vehicle access and walking for transport. Furthermore, our findings are in-line with previous research which has shown that elderly Japanese adults without a motor vehicle in the home were 1.43 times more likely to walk for transport than those with one or more motor vehicles. However, motor vehicle access was not associated with recreational walking in the Japanese study [29].

It is therefore plausible that the reason for increased transport-related walking in the LSA residents might be due limited options having no other option when they need to get from place to place. On the other hand, leisure time physical activity, which is more dependent on discretionary time and activity, may be lower in the LSA groups, in part, in compensation for the transport-related activity, or perhaps due to less discretionary time, as a consequence of active transport. Socio-economic status might be another reason for the differences in leisure-time physical activity between the HAS and LSA. Previous research has shown that individuals from lower socio-economic and minority groups are less likely to participate in leisure-time physical activity than those from higher socio-economic groups [30].

The number of destinations in the two neighborhoods in our study, quantified as the NEWS Land-use Mix Diversity score, was similar for LSA and HSA. This means that the distances of stores and facilities such as the post office, banks and restaurants from the participants’ homes were similar. However, the NEWS Land-use Mix Access score, which is based on a composite score according the ability to access services and destinations within 10–15 min walk from the home, was significantly higher in LSA. Therefore, the ease of access to destinations and services in LSA might have also contributed to their increased levels of active transport. Indeed, previous research has shown that the ability to walk for transport might also be influenced by the availability and accessibility to destinations [31]. Nathan and colleagues reported that even after adjusting for demographic variables, older adults were more likely to walk if general services like the hairdresser or pharmacy were within 400 m or 800 m of the house [32].

The second important finding in our study was that the NEWS Land-use Mix Access score was negatively associated with leisure-time. Thus, the more services within 10–15 min from the home, the less time the older adults in our study spent in leisure-time physical activity. This is contrary to what we would have expected, as previous research suggests that destinations has a positive influence on physical activity [15]. Our finding is supported by Hanibuchi and colleagues who found that the number of destinations within a one-kilometer radius from an older adult’s house was negatively associated with total walking time [10]. These researchers quantified total walking time and did not record whether the walking was for transport or leisure [10]. Conversely, access to exercise and general facilities were positively associated with total neighborhood walking in Japanese and Australian older adults [29, 32]. The difference between these findings and those that we observed in our study might be due to the differences in research methodology. The NEWS instrument used in our study has a composite score for access to services while those used in the Japanese and Australian study looked at individual services. Those researchers disaggregated the various services as they postulated that different destinations are important to older adults. For example, older adults might be more likely to walk to healthcare services and less likely to walk to schools [32]. Moreover, findings from a recent research study shows that older adults are 1.19 times more likely to walk to venues that encourage social interaction like places of worship and restaurants [32]. None of the sub-questions in the NEWS Land-use Mix Access section included questions on access to healthcare facilities or restaurants or places of worship.

Furthermore, the NEWS Land-use Mix Diversity score, which is based on the proximity of various services, in our study was not associated with habitual levels of physical activity. In addition, Nathan et al. and Inoue et al. only measured walking, whereas our study included all physical activity. Therefore, we might have found significant associations if we disaggregated each of the questions used in the various NEWS scores or considered walking as the only form of physical activity.

Another finding from our study was total weekly MVPA was inversely associated with perceived safety from traffic. Motor vehicle accidents involving pedestrian deaths has been previously identified as one of the leading cause of non-natural death among older adults [14]. Thus, it is reasonable to expect that issues of safety from traffic will contribute to habitual levels of physical activity, especially in older adults [33]. Indeed, the older adults participating in the study by Tsunoda and co-workers were more likely to walk for at least one hour per week if there were good traffic safety measures and pleasing neighborhood aesthetics [34]. The older adults in their study were more likely to meet the physical activity guidelines if sidewalks were present [34].

Similarly, our results shows that the availability of walk and cycle paths were positively associated with transport-related physical activity for LSA residents. Likewise, older adults in Hong Kong were more likely to participate in recreational walking if the infrastructure included bridges and connecting services was present [35]. These findings, together with ours, underscore the role of the built environment attributes to promote walking in older adults.

As expected, the older adults living in HSA found their neighborhood more aesthetically pleasing than the LSA residents. Leisure time physical activity was higher the more aesthetically pleasing the residents’ perceptions of the neighborhood. This relationship was also present for those in HSA. This finding is in line with those from other studies, including one among Japanese older adults [34]. Furthermore, the older adults in HSA reported that they always use their motor vehicles when travelling from place to place, they may have chosen to be physically active in another neighborhood or facility. Based on our study design, most of the older adults were recruited from a commercial gym and seniors club, and this could be where they participate in most of their leisure time and MVPA.

### Limitations and strengths

This is a pilot study and we employed a convenience sampling strategy, with only those living within a 5 km radius to the commercial gym or community-based exercise club being eligible to participate. As a result, with only 44 participants, this reduces the generalizability of our findings. Another limitation is that because our participants were recruited from exercise groups, they might be more active than other older adults. Nevertheless, this is the first study to investigate the role of the built environment and physical activity in older adults in the Global South Our findings do provide some baseline data and could contribute to future research that will include larger numbers of participants.

The strengths of this research study include the use of both self-report and objective measures of physical activity. Previous research has shown that participants over-estimate physical activity in surveys, whereas objective measures like the accelerometer, might under-estimate total physical activity. Furthermore, we were able to disaggregate the various intensities of physical activity and could calculate transport-related physical activity.

## Conclusion

Data from this pilot study shows that older adults living in lower socio-economic status neighborhoods were more likely to participate in transport related physical activity than those from higher income neighborhoods. Built environment attributes associated with access to services were negatively associated with leisure-time and total MVPA. Factors such as motor vehicle ownership and self-perceived health status might have contributed the findings observed in our study.

These findings provide some preliminary data to support the provision of traffic safety measures in order to promote physical activity in older adults. In addition, promoting leisure time physical activity and transport-related activity among older adults living in LSA and HSA, respectively. Further African-based research with larger sample sizes in this age group are therefore required to provide more comprehensive and conclusive evidence on the role of the built environment and physical activity in older adults.

## References

[1] Steyn KFJ, Temple N, Steyn KFJ, Temple N (2006). Chronic Diseases of Lifestyle in South Africa: 1995–2005. Technical Report. Cape Town: South African Medical Research Council.

[2] Mayosi BM, Flisher AJ, Lalloo UG, Sitas F, Tollman SM, Bradshaw D (2009). The burden of non-communicable diseases in South Africa. Lancet.

[3] Phaswana-Mafuya N, Peltzer K, Chirinda W, Musekiwa A, Kose Z, Hoosain E (2013). Self-reported prevalence of chronic non-communicable diseases and associated factors among older adults in South Africa. Global Health Action.

[4] Lee IM, Shiroma EJ, Lobelo F, Puska P, Blair SN, Katzmarzyk PT (2012). Effect of physical inactivity on major non-communicable diseases worldwide: an analysis of burden of disease and life expectancy. Lancet.

[5] Hubbard RE, Fallah N, Searle SD, Mitnitski A, Rockwood K (2009). Impact of exercise in community-dwelling older adults. PLoS One.

[6] Schutzer KA, Graves BS (2004). Barriers and motivations to exercise in older adults. Prev Med.

[7] Warburton DE, Nicol CW, Bredin SS (2006). Health benefits of physical activity: the evidence. CMAJ.

[8] Alburquerque-Sendin F, Barberio-Mariano E, Brandao-Santana N, Rebelatto DA, Rebelatto JR (2012). Effects of an adapted physical activity program on the physical condition of elderly women: an analysis of efficiency. Rev Bras Fisioter.

[9] Chodzko-Zajko WJ, Proctor DN, Fiatarone Singh MA, Minson CT, Nigg CR, Salem GJ (2009). American College of Sports Medicine position stand. Exercise and physical activity for older adults. Med Sci Sports Exerc.

[10] Hanibuchi T, Kawachi I, Nakaya T, Hirai H, Kondo K (2011). Neighborhood built environment and physical activity of Japanese older adults: results from the Aichi Gerontological Evaluation Study (AGES). BMC Public Health.

[11] Guthold R, Ono T, Strong KL, Chatterji S, Morabia A (2008). Worldwide variability in physical inactivity a 51-country survey. Am J Prev Med.

[12] Shisana OL D, Rehle T, Simbayi L, Zuma K, Dhansay A, Reddy P (2013). The South African National Health and Nutrition Examination Survey: SANHANES-1. SANHANES-1.

[13] Sharpe PA, Jackson KL, White C, Vaca VL, Hickey T, Gu J (1997). Effects of a one-year physical activity intervention for older adults at congregate nutrition sites. Gerontologist.

[14] Amosun SL, Burgess T, Groeneveldt L, Hodgson T (2007). Are elderly pedestrians allowed enough time at pedestrian crossings in Cape Town, South Africa?. Physiother Theory Pract.

[15] Owen N, Humpel N, Leslie E, Bauman A, Sallis JF (2004). Understanding environmental influences on walking; Review and research agenda. Am J Prev Med.

[16] Sallis JF, Bowles HR, Bauman A, Ainsworth BE, Bull FC, Craig CL (2009). Neighborhood environments and physical activity among adults in 11 countries. Am J Prev Med.

[17] Gomez LF, Parra DC, Buchner D, Brownson RC, Sarmiento OL, Pinzon JD (2010). Built environment attributes and walking patterns among the elderly population in Bogota. Am J Prev Med.

[18] Siqueira Reis R, Hino AA, Ricardo Rech C, Kerr J, Curi Hallal P (2013). Walkability and physical activity: findings from Curitiba, Brazil. Am J Prev Med.

[19] 2011 Census Suburb Profiles**.** [http://www.capetown.gov.za/en/stats/Pages/2011-Census-Suburb-Profiles-land.aspx]

[20] Kolbe-Alexander TL, Lambert EV, Charlton KE (2006). Effectiveness of a community based low intensity exercise program for older adults. J Nutr Health Aging.

[21] Clark P. Public transport in metropolitan Cape Town: Past, present and future. *Transport* Reviews;22(1):77–101.

[22] Cerin E, Conway TL, Saelens BE, Frank LD, Sallis JF (2009). Cross-validation of the factorial structure of the Neighborhood Environment Walkability Scale (NEWS) and its abbreviated form (NEWS-A). Int J Behavioral Nutrition and Physical Activity.

[23] Cerin E, Conway TL, Cain KL, Kerr J, De Bourdeaudhuij I, Owen N (2013). Sharing good NEWS across the world: developing comparable scores across 12 countries for the Neighborhood Environment Walkability Scale (NEWS). BMC Public Health.

[24] Bull FC, Maslin TS, Armstrong T (2009). Global physical activity questionnaire (GPAQ): nine country reliability and validity study. J Phys Act Health.

[25] Matthews CE (2005). Calibration of accelerometer output for adults. Med Sci Sports Exerc.

[26] Gorman E, Hanson HM, Yang PH, Khan KM, Liu-Ambrose T, Ashe MC (2014). Accelerometry analysis of physical activity and sedentary behavior in older adults: a systematic review and data analysis. Eur Rev Aging Phys Act.

[27] Cerin E, Mellecker R, Macfarlane DJ, Barnett A, Cheung MC, Sit CH (2013). Socioeconomic status, neighborhood characteristics, and walking within the neighborhood among older Hong Kong Chinese. J Aging Health.

[28] Turrell G, Haynes M, Wilson LA, Giles-Corti B (2013). Can the built environment reduce health inequalities? A study of neighbourhood socioeconomic disadvantage and walking for transport. Health Place.

[29] Inoue S, Ohya Y, Odagiri Y, Takamiya T, Kamada M, Okada S (2011). Perceived neighborhood environment and walking for specific purposes among elderly Japanese. J Epidemiol.

[30] King AC (2001). Interventions to promote physical activity by older adults. J Gerontol A Biol Sci Med Sci.

[31] Cerin E, Lee KY, Barnett A, Sit CH, Cheung MC, Chan WM (2013). Walking for transportation in Hong Kong Chinese urban elders: a cross-sectional study on what destinations matter and when. Int J Behavioral Nutrition Physical Activity.

[32] Nathan A, Pereira G, Foster S, Hooper P, Saarloos D, Giles-Corti B (2012). Access to commercial destinations within the neighbourhood and walking among Australian older adults. Int J Behavioral Nutrition and Physical Activity.

[33] DiPietro L (2001). Physical activity in aging: changes in patterns and their relationship to health and function. J Gerontol A Biol Sci Med Sci.

[34] Tsunoda K, Tsuji T, Kitano N, Mitsuishi Y, Yoon JY, Yoon J (2012). Associations of physical activity with neighborhood environments and transportation modes in older Japanese adults. Prev Med.

[35] Cerin E, Sit CH, Barnett A, Cheung MC, Chan WM (2013). Walking for recreation and perceptions of the neighborhood environment in older Chinese urban dwellers. J Urban Health.

